# Preliminary Studies on the Effects of Oyster Mushroom Spherical Virus China Strain on the Mycelial Growth and Fruiting Body Yield of the Edible Mushroom *Pleurotus ostreatus*

**DOI:** 10.3390/biology11040574

**Published:** 2022-04-10

**Authors:** Hai-Jing Hu, Jian-Rui Wang, Xian-Hao Cheng, Yu Liu, Xiao-Yan Zhang

**Affiliations:** Shandong Key Laboratory of Edible Mushroom Technology, School of Agriculture, Ludong University, Yantai 264025, China; haijing975@163.com (H.-J.H.); jianrui302@163.com (J.-R.W.); chengxianhao@sohu.com (X.-H.C.); liuyu8006@163.com (Y.L.)

**Keywords:** oyster mushroom spherical virus, genome sequence, phylogenetic analysis, virus curing, *Pleurotus ostreatus*

## Abstract

**Simple Summary:**

Oyster mushroom spherical virus (OMSV) is a positive-sense single-stranded RNA mycovirus which is associated with a devastating oyster mushroom die-back disease. However, little is known about its classification, and the effects of OMSV infection on its fungal host remain unclear. In the present study, we present the molecular evidence that virus isolates from the *P. ostreatus* 8129 strain in China represent a new strain of OMSV, named OMSV-Ch. Phylogenetic analysis based on the putative replication protein (RP) suggest that the OMSV may belong to a new yet-to-be-established genus of the *Tymoviridae*. Using single hyphal tip cultures combined with high-temperature treatment, we obtained the OMSV-Ch-cured *P. ostreatus* strain. Preliminary studies indicate that OMSV-Ch infection can significantly inhibit mycelial growth, cause malformation symptoms, and reduce the yield of the fruiting bodies of the edible mushroom *P. ostreatus*. Furthermore, OMSV-Ch can horizontally transfer to a virus-cured strain.

**Abstract:**

Oyster mushroom spherical virus (OMSV) is a positive-sense single-stranded RNA mycovirus which is associated with a devastating oyster mushroom die-back disease. However, little is known about its diversity, and the effects of OMSV infection on its fungal host are not well understood. In this study, we determined the nearly complete nucleotide sequence of OMSV isolated from cultivated oyster mushrooms in China. Sequence analysis suggested that the virus represents a new strain of OMSV (referred to here as OMSV-Ch). A GenBank BLAST search of the genomic sequences demonstrated that the OMSV-Ch had the highest identity (74.9%) with the OMSV from Korea (OMSV-Kr). At the amino acid–sequence level, these two strains shared 84.1% identity in putative replication protein (RP) and 94.1% identity in coat protein (CP). Phylogenetic analysis based on RP showed that OMSV-Ch clustered with OMSV-Kr, closely related to *Tymoviridae*. Phylogenetic analysis based on both the RP and CP showed that OMSV had a distant clade relationship with tymoviruses, marafiviruses, and maculaviruses. We obtained the OMSV-Ch-free *Pleurotus ostreatus* strain via single hyphal tip cultures combined with high-temperature treatment. Preliminary studies indicate that OMSV-Ch can significantly inhibit mycelial growth, cause malformations of the fruiting bodies, and reduce the yield of *P. ostreatus*. Co-cultivation resulted in horizontal transmission of the OMSV-Ch to a virus-cured strain. The findings of our study contribute to the prevention and control of mycoviral diseases in the future.

## 1. Introduction

Mycoviruses, also known as fungal viruses, are a group of viruses that naturally infect fungi, including plant pathogenic fungi and mushrooms. The majority of mycoviruses contain double-stranded RNA (dsRNA) genomes, but a large number of positive sense (+) or negative sense (−) single-stranded RNA (ssRNA) and DNA (ssDNA) mycoviruses have also been discovered [1]. Most mycovirus infections are cryptic or latent with no obvious symptoms on the hosts, while some mycoviruses cause significant effects on host growth, development, reproduction, and virulence [2,3,4,5]. According to the current literature, mycoviruses have been isolated and characterized in nine different cultivated edible macrofungal species, including *Agaricus bisporus*, *Pleurotus ostreatus*, *Flammulina velutipes*, *Lentinula edodes*, *Cyclocybe aegerita*, *Boletus edulis*, *Volvariella volvacea*, *Armillaria*, and *Grifola frondosa* [6,7,8,9,10,11,12,13,14,15,16,17,18]. The mycovirus infection causes various symptoms in mushrooms, including retarded mycelial growth, delayed fruiting body development, and deformed fruiting bodies, which often leads to a significant reduction in the yield and commercial value of mushroom products [19,20].

*Pleurotus ostreatus* is an edible oyster mushroom cultivated throughout the world with high economic, nutritional, and medicinal values [21,22]. Several mycoviruses have been reported to infect *P. ostreatus*, including dsRNA viruses such as oyster mushroom isometric virus (OMIV) I and II, *P. ostreatus* virus 1 (PoV1), *P. ostreatus* spherical virus (POSV), *P. ostreatus* virus ASI2792 (PoV-ASI2792), *P. ostreatus* virus Shin-Nong (PoV-SN), and (+)ssRNA-virus oyster mushroom spherical virus (OMSV) [11,14,23,24,25,26]. Oyster mushrooms infected with different mycoviruses exhibit different morphological and physiological characteristics. The OMIV-infected *P. ostreatus* showed abnormal phenotypic characteristics including retarded mycelial growth, undeveloped stipes, and deformed caps on the fruiting bodies. By contrast, infection with PoV1 in *P. ostreatus* showed normal morphological and growth phenotypes [14,25]. Studies have shown that infection with POSV and other mycoviruses in the *P. ostreatus* TD300 strain is the cause of mushroom-strain degeneration [11]. A previous study by Song et al. demonstrated that infection with PoV-ASI2792 has a deleterious effect on the vegetative growth of *P. ostreatus* [23].

OMSV was first identified in 2003 from cultivated oyster mushrooms in Korea. It is a spherical virus 27 nm in diameter and contains a monopartite positive-sense ssRNA genome 5784 bp in size and consisting of seven open reading frames (ORFs). ORF1 encodes a protein with the RNA-dependent RNA polymerase (RdRP) and helicase motifs. ORF2 encodes a 28.5 kDa coat protein. ORFs 3–6 are located within the ORF1 sequence encoding the putative polypeptides that are 12, 12.5, 21, and 14.5 kDa, respectively. ORF7 overlaps with ORF2 and encodes a 23 kDa protein. However, the function of the predicted proteins encoded by ORFs 3–7 remains unknown. The 5′ untranslated region (UTR) consists of 78 nt and is located upstream of the ORF1, and the 3′ UTR is comprised of 99 nt, including the 74 nt poly (A) tail at the 3′ end. The genome organization and amino acid sequence analysis of the RdRP and helicase motifs shows that OMSV is most closely related to tymoviruses. OMSV is associated with a devastating oyster mushroom die-back disease and malformed oyster mushrooms are frequently associated with OMSV infection in the suburbs of Beijing, China [27]. A technique using a surface plasmon resonance biosensor chip combined with a triple antibody sandwich enzyme-linked immunosorbent assay (TAS-ELISA) was previously developed to rapidly detect OMSV infection [14]. As mixed infections with two or more mycoviruses are common, the co-infection of OMSV and OMIV in *P. ostreatus* cv. Chunchu in the southern regions of Korea has been reported and often results in the harvest losses [20].

In this study, we obtained and characterized an OMSV China strain (OMSV-Ch) from cultivated *P. ostreatus*. Phylogenetic analyses revealed that the OMSV-Ch and OMSV Korea (OMSV-Kr) strains are related to members of the order *Tymovirales* but placed in a clade distinct from those formed by other *Tymovirales* members. We obtained an OMSV-Ch-cured strain and compared it with the OMSV-Ch-infected original strain in mycelial growth and fruiting body yield. Furthermore, we introduced the OMSV-Ch into the virus-cured subculture via co-culture. The findings of our study contribute to the prevention and control of mycoviral diseases in the future.

## 2. Materials and Methods

### 2.1. Mushroom Strain

The malformed fruiting bodies of *P. ostreatus* strain 8129 were collected from a commercial mushroom farm in Yantai City, Shandong Province, China.

### 2.2. RNA Extraction and Reverse Transcription PCR

Total RNA was extracted from mycelia or fruiting bodies of *P. ostreatus* using RNA Easy Fast Plant Tissue Kit (Tiangen, Beijing, China). OMSV was detected using two pairs of primers, OMSV-4959F/OMSV-5605R or OMSV-CPF/OMSV-CPR (Supplementary Table S1). First-strand complementary DNA (cDNA) was synthesized from 2 µg total RNA using a reverse primer and M-MLV reverse transcriptase (Promega). The reverse transcription (RT) reaction was performed in a 15 μL PCR mixture consisting of 3 μL 5 × RT buffer, 0.5 μL reverse primer (10 uM), 5 μL RNase-free ddH_2_O, 1 μL dNTP mixture (each 2.5 mM), 0.25 μL RNase inhibitor (40 U/μL), 0.25 μL MLV reverse transcriptase (200 U/μL) and 5 μL plant total RNA (~2000 ng). The RT reaction was incubated for 90 min at 37 °C. PCR for detection of OMSV was carried out in a 15 μL mixture containing 7.5 μL 2 × Taq PCR MasterMix II (Tiangen, Beijing, China), 2 μL cDNA template, 0.5 μL of specific primers (10 μM) (Supplementary Table S1), and 4.5 μL ddH_2_O. After PCR amplification, the products were analyzed by 1% agarose gel electrophoresis and stained with ethidium bromide.

For quantification of OMSV RNA accumulation, semi-quantitative RT-PCR was performed. Total RNA was isolated from the mycelia or fruiting bodies. After measuring the total amount of RNA (2000 ng), cDNAs were synthesized from total RNA using M-MLV reverse transcriptase (Promega). Primers targeting OMSV RNA genomes are described in Table S1. Actin was used as an internal control by using the primers actin-F/actin-R [28].

### 2.3. Cloning and Sequencing

To determine the genome sequence of OMSV China strain, RT reaction products were amplified with primers OMSV-5F/OMSV-2888R or OMSV-2821F/OMSV-3R by using the TIANSeq HiFi Amplification Mix (Tiangen, Beijing, China). After adding adenine, purified PCR products were inserted into pMD19-T vectors and then transformed into *Escherichia coli* DH5α-competent cells. Three positive clones were selected for sequencing (Sangon, Shanghai, China).

### 2.4. Phylogenetic Tree Construction

The viral genome sequences and amino acid sequences were obtained from the NCBI (National Center for Biotechnology Information) database. The phylogenetic trees were constructed according to the neighbor-joining method with 1000 bootstrap replicates by using the MEGA 7.0 program.

### 2.5. Virus Curing

To cure the OMSV-Ch-infected *P. ostreatus* strain, the mycelia were isolated from the OMSV-infected mushroom tissue and cultured on potato dextrose agar (PDA) amended with 50 μg/mL ampicillin (Amp) and incubated at 32 °C for 7 days in darkness. Mycelia disk with a diameter of 7.5 mm was taken out from the PDA plate and inoculated onto a new agar plate which had no nutrients. The agar plates were incubated at 32 °C in the dark for 6 days. Then hyphal tip with a diameter of 2–3 mm was cut from the growing edge of the plate and transferred to a PDA + Amp plate. The transfer of hyphal tip to new plates was repeated successively until the virus was completely eliminated.

### 2.6. Determination of Growth Rate

The growth rate of virus-infected *P. ostreatus* was compared with virus-cured lines using PDA. The mycelium block (7.5 mm diameter) was obtained with a puncher on the activated mycelia plate and then inoculated on the prepared PDA, which was cultured at 25 °C in the dark. The center of the mycelium block was chosen as the cross point to draw a vertical line. The growth length of mycelium was recorded every day until the colony diameters covered more than 90% of the plate, and then the average growth speed of mycelium was calculated. All measurements of the growth rate on culture plates were performed with at least three biological and three technical replicates.

### 2.7. Cultivation Tests

For spawn preparation, each inoculum of the *P. ostreatus* strain was grown in potato-dextrose broth (PDB) at 25 °C in the dark for 7 days in a Erlenmeyer flask with continuous agitation at 150 rpm. The mycelium was grown in bags which contained cotton-seed hull and wheat bran. Cotton-seed hull, wheat bran, and lime were mixed at a ratio of 80:18:2 and the mixture was adjusted to a water content of 60% in the bag. The wetted substrates, weighing 1.5 kg, were placed in the polypropylene bags and sterilized at 121 °C for 2 h. Each bag was inoculated with mycelia grown in PDB and incubated in the dark at 25 ± 1 °C. After the surface of the substrate was completely covered with mycelia, the bags were transferred to horizontal racks in a cropping room at 22 ± 1 °C and 85–90% relative humidity under semidarkness. After primordia developed, the bags were opened with a blade. Adequate ventilation was provided to prevent increase in CO_2_ concentration. The bags were watered twice a day during cropping.

Fruiting bodies were harvested from the substrates manually 7 days after primordia initiation. The yield of oyster mushroom was determined by measuring the weight per bag. Statistical analysis was performed using a Student’s *t* test. Values were considered significant when the *p* value was 0.05 or less.

### 2.8. Horizontal OMSV-Ch Transmission

To test whether horizontal transmission of OMSV-Ch occurs from an OMSV-Ch-infected donor strain to a cured recipient strain, the OMSV-Ch-infected and -cured strains were co-cultivated on PDA at 25 °C for 5 days. During co-cultivation experiments, cultures were monitored and the hyphae were extracted when the donor and recipient strains made contact. One inoculum from the donor side (I) and two from the recipient (II and III) were sub-cultivated for 5 days, and the presence of OMSV was tested by RT-PCR. At the same time, the growth rate of the mycelia was measured and statistical significance was assessed by *t* test.

## 3. Results

### 3.1. Nucleotide Sequence and Genome Organization of the OMSV-Ch Strain

**Figure 1:**
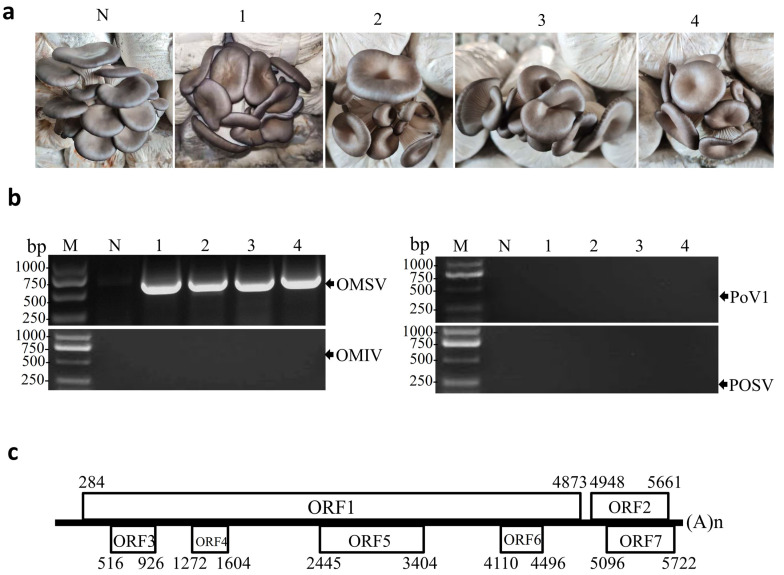
RT-PCR detection of OMSV in malformed fruiting bodies of *P. ostreatus*. (**a**) The collected fruiting body samples from *P. ostreatus* cultivar 8129 display symptoms of funnel-shaped or morning glory–shaped caps. (**b**) RT-PCR detection for the presence or absence of OMSV, OMIV, POSV, or PoV1 from fruiting body samples described in (**a**). Lanes 1–4, four fruiting body samples of *P. ostreatus* that were positive for OMSV by RT-PCR. N, negative control, the healthy *P. ostreatus* sample; M, GL DNA Marker2000. (**c**) Schematic representation of the OMSV-Ch genome structure. Boxes represent the ORFs. Numbers represent nucleotide positions.

In December 2019, samples from *P. ostreatus* (8129 strain) fruiting bodies with severe malformations were collected from commercial mushroom farms in Yantai City, Shandong Province, China. Four samples displayed deformed morphologies with funnel-shaped or morning glory–shaped caps (Figure 1a). Total RNA was extracted from the fruiting bodies and examined by reverse transcriptase-PCR (RT-PCR) with specific primers for OMIV, POSV, PoV1, and OMSV. None of the four samples were positive for OMIV, POSV, or PoV1 (Figure 1b). In contrast, fragments of the expected size (750 bp) for OMSV were obtained and the sequencing result confirmed that the collected fruiting bodies were infected with OMSV (Figure 1b). Since no complete genomic sequence of OMSV infecting *P. ostreatus* in China had been previously reported, we analyzed the genomic sequence of the OMSV China strain. Primers for RT-PCR amplification of the genome sequences were derived from the OMSV-Kr and the primer sequences are presented in Table S1. Two overlapping genomic fragments (OMSV-5F/OMSV-2888R and OMSV-2821F/OMSV-3R) were obtained by RT-PCR and cloned. At least three independent, positive PCR clones were sequenced. Thus, except for the short region where the primers annealed at the 5′ and 3′ termini, the nearly complete genomic sequence of the OMSV-Ch strain 5747 nt in size was determined and given the GenBank accession number OL546221. A GenBank BLAST search of the genomic sequences showed that OMSV-Ch had the highest identity (74.9%) with OMSV-Kr (GenBank NC_004560.1). The two strains shared 72.4 and 84.1% nucleotide and amino acid–sequence identity in RP, respectively, while OMSV-Ch shared nucleotide (90.6%) and amino acid–sequence identity (94.1%) with the OMSV-Kr strain in CP.

Analysis of the nearly complete genomic sequence of OMSV-Ch was conducted using DNAMAN software (Version 8.0, Lynnon Biosoft, Canada). The OMSV-Ch genome exhibited a high cytosine proportion and low adenine proportion (18.1% A; 31.3% C; 24.2% G; 26.4% T). The genome contained a 5′ UTR, 3′ UTR, an intergenic non-coding region (intergenic NCR), and seven ORFs which encoded polypeptides that were greater than 10 kDa in size (Figure 1c). The 5′ UTR was 283 nt in length, much longer than the corresponding regions in the OMSV-Kr strain (171 nt). The first ORF (ORF1), beginning with an AUG from nt 284 to 286 ended with a UAA from nt 4871 to 4873. The ORF1 produced an RP of about 168.9 kDa in molecular weight, smaller than that in OMSV-Kr (about 174 kDa). The predicted RP contained the highly conserved glycine/aspartic acid/aspartic acid (GDD) sequence motif at amino acid (aa) position 1363–1365, which is typically found in positive-strand RNA viruses [29]. A conserved helicase sequence, GTAGCGKS, in the predicted RP was also detected at aa position 690–697. ORF2, located at the 3′ region of the RNA, consisted of 714 nt (236 aa) and encoded a predicted CP with a molecular weight of 24.0 kDa (Figure 1c). ORF1 and ORF2 were separated by a 74 nt intergenic NCR (from nt 4874 to 4947), which was slightly shorter than that in OMSV-Kr (77 nt in length). Five other ORFs were also predicted in ORF3, 4, 5, and 6, and were located within the ORF1 sequence encoding the putative polyproteins with molecular weights of 14.5, 11.6, 34.5, and 13.3 kDa, respectively. ORF7 (from nt 5096 to 5722) was predicted to encode a 22.0 kDa protein, which overlapped with ORF2 (Figure 1c). The nucleotide sequence identities between the OMSV-Ch and OMSV-Kr strains were 73.2% for ORF3, 42.3% for ORF4, 40.8% for ORF5, 83.6% for ORF6, and 88.4% for ORF7. At the amino acid–sequence level, the OMSV-Ch and OMSV-Kr strains shared 38.1% in ORF3, 23.4% in ORF4, 17.4% in ORF5, 58.6% in ORF6, 75.7% in ORF7. The two OMSV strains had greater divergence in 5′-terminal ORFs 1, 3, 4, 5, and 6 than in 3′-terminal ORFs 2 and 7. The function of the proteins encoded by ORF3 to ORF7 remains unknown.

### 3.2. Phylogenetic Analysis of OMSV-Ch

**Figure 2:**
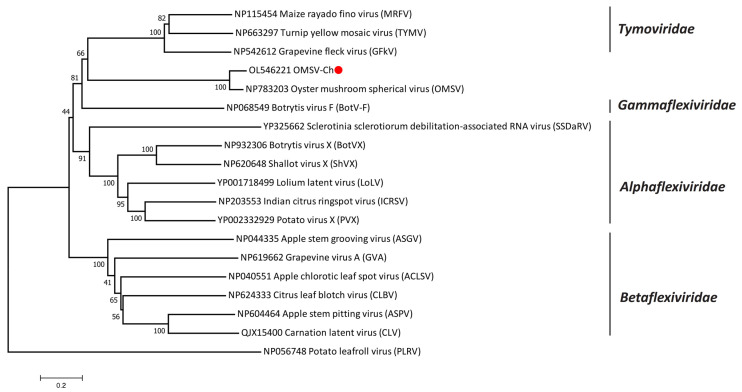
Phylogenetic tree based on the amino acid sequences of the replication protein of OMSV-Ch and 17 representatives of the order *Tymovirales*. The OMSV-Ch sequence from the present study is marked with a red dot. Potato leafroll virus (PLRV) was used as an outgroup.

**Figure 3:**
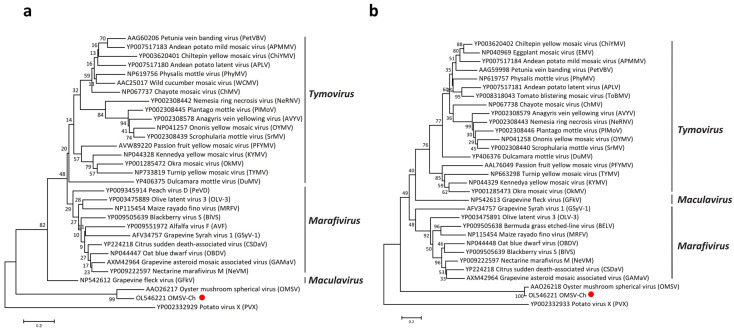
Phylogenetic analysis of the OMSV-Ch replication protein (**a**) and coat protein (**b**) with corresponding proteins of tymoviruses, marafiviruses, and maculaviruses. The OMSV-Ch sequence from the present study is marked with a red dot. Potato virus X (PVX) was used as an outgroup.

According to the International Committee on Taxonomy of Viruses (ICTV), OMSV is considered an unclassified virus, though it has been previously reported to have the closest relationship with tymoviruses [26]. In order to better understand the taxonomic status of OMSV, phylogenetic trees were generated and visualized with MEGA (Version 7.0). A phylogenetic tree based on the RP amino acid sequences of the members in the order *Tymovirales* showed that the OMSV-Ch strain clustered with OMSV-Kr and that they were both more closely related to the *Tymoviridae* than to any other family (*Alphaflexiviridae*, *Betaflexiviridae*, or *Gammaflexiviridae*) of the *Tymovirales* order (Figure 2). Further phylogenetic analysis based on the RP and CP showed that the OMSV was distinct from tymoviruses, marafiviruses, and maculaviruses (Figure 3a,b), suggesting that the OMSV may belong to a new genus of the *Tymoviridae* family.

### 3.3. Curing of OMSV-Ch from P. ostreatus

**Figure 4:**
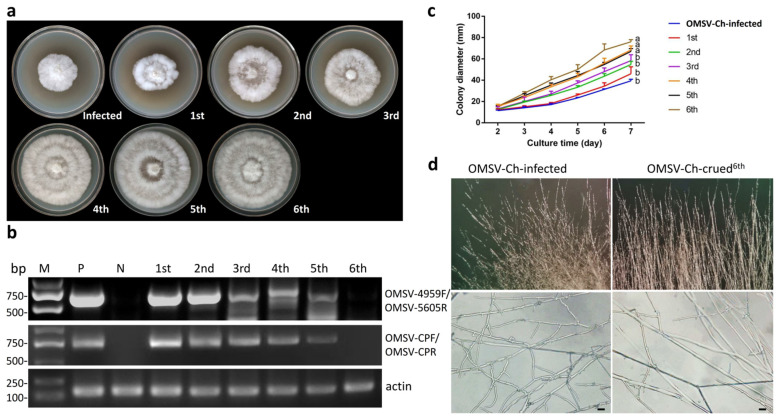
Curing of OMSV-Ch of *P. ostreatus* on PDA plates. (**a**) Colony morphology of the OMSV-Ch-infected strain compared with the OMSV-Ch-cured strain after 7 days of incubation at 25 °C. (**b**) Semi-quantitative RT-PCR detection of OMSV-Ch accumulation from each generation. The primers OMSV-4959F/OMSV-5605R (upper panel) and OMSV-CPF/OMSV-CPR (middle panel) were used for RT-PCR detection of OMSV-Ch. Actin served as an internal control (lower panel). N, negative control, the healthy *P. ostreatus* sample; P, positive control, the OMSV-Ch-infected *P. ostreatus* sample; M, GL DNA Marker2000. (**c**) Effects of OMSV-Ch curing on mycelial growth. Values are significantly different at *p* ≤ 0.05, by Student’s *t* test. (**d**) Microscopic observation of the colony margin in the OMSV-Ch-infected strain compared with the OMSV-Ch-cured strain. The fungal hyphae were observed at 400× magnification (lower panel). Bars = 20 μm.

We determined that the mycelia of the *P. ostreatus* 8129 strain were infected with OMSV via RT-PCR. In order to better understand the effect of OMSV-Ch infection on the vegetative characteristics of *P. ostreatus*, we aimed to cure the *P. ostreatus* 8129 from the OMSV-Ch in order to obtain an OMSV-Ch-cured isogenic strain. The single hyphal tip culture method combined with heat treatment, as described in the “Materials and Methods” section, was used for OMSV-Ch curing. The mycelia were transferred to potato dextrose agar (PDA) containing 50 μg/mL ampicillin and incubated at 32 °C, and this process was repeated 6 times. In order to examine the effects of the OMSV-Ch infection on the colony growth, the isogenic OMSV-Ch-cured and OMSV-Ch-infected *P. ostreatus* strains were cultured on PDA plates. After 7 days of culture, we observed the mycelial growth displayed an accelerated growth rate along with the increasing number of transfer treatments (Figure 4a). The presence or absence of the OMSV-Ch in the *P. ostreatus* mycelia was determined by RT-PCR detection with two pairs of primers. The sixth generation of the mycelia became free of OMSV-Ch (Figure 4b).

To further confirm this result, we measured the mycelial growth rate by monitoring the diameter of the colony every other day for 7 consecutive days. The result showed that the OMSV-Ch-infected strain displayed a slower growth rate than the OMSV-Ch-cured strain. The mycelial growth rate in the OMSV-Ch-cured strain (sixth generation) was approximately 1.65 times higher compared with the OMSV-Ch-infected strain at 7 days after inoculation (Figure 4c). Microscopic observation of the colony morphology of the marginal hyphae showed that the OMSV-Ch-infected strain often had an irregular margin that differed from the OMSV-Ch-cured strain (Figure 4d). Furthermore, the diameter of the OMSV-Ch-infected mycelia was 0.63 times smaller than that of the OMSV-Ch-cured mycelia (Figure S1). These results indicate that infection of OMSV-Ch inhibits the mycelial growth of *P. ostreatus*.

### 3.4. Effects of OMSV-Ch on the Phenotype and Yield of the Fruiting Bodies

**Figure 5:**
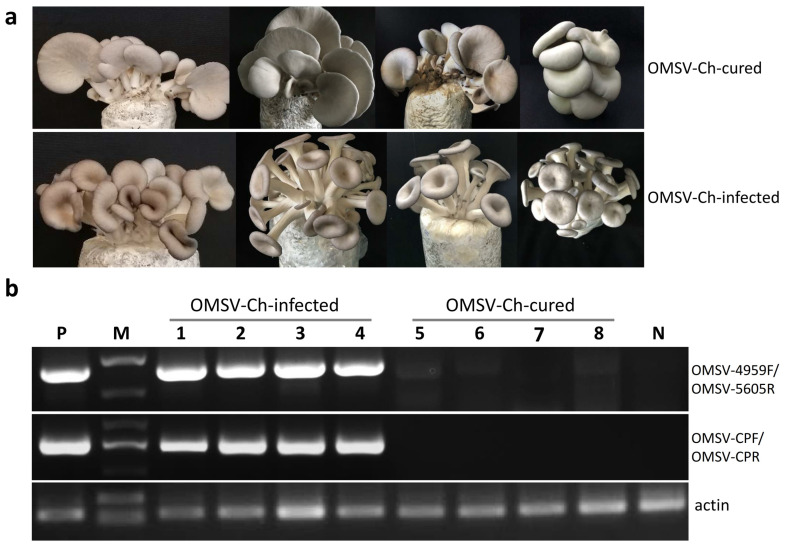
The cultivation test to examine the effects of the OMSV-Ch infection on *P. ostreatus* fruiting bodies. (**a**) The morphological characteristics of fruiting bodies of the OMSV-Ch-cured (upper panel) and OMSV-Ch-infected (lower panel) strains. (**b**) Semi-quantitative RT-PCR analysis for OMSV-Ch in fruiting bodies of OMSV-Ch-infected and OMSV-Ch-cured strains. Different bags of fruiting bodies were collected from the OMSV-Ch-infected (lanes 1–4) and OMSV-Ch-cured strains (lanes 5–8). The primers OMSV-4959F/OMSV-5605R (upper panel) and OMSV-CPF/ OMSV-CPR (middle panel) were used for RT-PCR detection of OMSV-Ch. Actin served as an internal control (lower panel). N, negative control, the healthy *P. ostreatus* sample; P, positive control, the OMSV-Ch-infected *P. ostreatus* sample; M, GL DNA Marker2000.

In order to investigate the effect of OMSV-Ch infection on the performance and yield of the fruiting bodies, the isogenic OMSV-Ch-cured and -infected *P. ostreatus* strains were subjected to cultivation tests. Since the OMSV-Ch-infected mycelia showed slower growth on PDA than the OMSV-Ch-cured mycelia, we compared the average cultivation period from inoculation with mycelia to harvest of the mushrooms from the first flush. The data showed that the average cultivation period of the virus-infected strain was six days longer than that of the virus-cured strain, suggesting that the mycelia of the virus-infected strain grew slower than that of the virus-cured strain (Table 1). We then observed the fruiting body morphology. At the first flush stage, fruiting bodies displayed an abnormal phenotype, including a bent, long, thin stipe with small caps, funnel-shaped or morning glory–shaped caps in the OMSV-Ch-infected strain. By contrast, the OMSV-Ch-cured strain fruiting bodies displayed a normal phenotype with a thick stipe and middle-sized cap (Figure 5a). In order to analyze the accumulation of OMSV-Ch in the mushroom, fruiting bodies were randomly collected and subjected to semi-quantitative RT-PCR for OMSV-Ch detection. RT-PCR detection showed that a high accumulation of OMSV-Ch RNAs was detected in the fruiting bodies of the OMSV-Ch-infected strain, while the OMSV-Ch RNAs were not detected in the OMSV-Ch-cured strain fruiting bodies (Figure 5b). When harvested, the first and second flushes of the fruiting bodies were collected to determine the average yield. By the second flush, the average yield of the OMSV-Ch-cured strain (389.44 g/bag) was significantly (*p* < 0.001) higher than that of the OMSV-Ch-infected strain (292.82 g/bag). In other words, the average yield of the OMSV-Ch-cured strain was 33% higher compared with that of the OMSV-Ch-infected strain (Table 1). Preliminary analysis of these results suggests that the OMSV-Ch infection causes the malformation symptoms as well as reduces the yield of *P. ostreatus* fruiting bodies.

### 3.5. OMSV-Ch Can Horizontally Transfer to a Virus-Cured Strain

**Figure 6:**
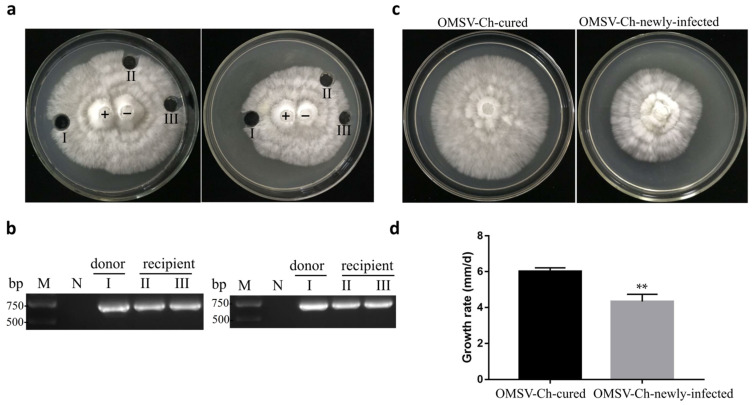
Pairwise co-cultivation of OMSV-Ch-infected (+) and OMSV-Ch-cured (−) strains of *P. ostreatus.* (**a**) After incubation for 5 days at 25 °C, one inoculum from the donor side (I) and two inocula from the recipient (II and III) were sub-cultivated for 5 days. (**b**) RT-PCR detection for the presence of OMSV. Negative controls of pairing tests were OMSV-Ch-cured strains. M, GL DNA Marker2000. (**c**) Colony morphology of the OMSV-Ch-cured strain compared to the newly infected OMSV-Ch strain after 5 days of incubation at 25 °C. (**d**) Growth rate of OMSV-Ch-cured and newly infected OMSV-Ch strains of *P. ostreatus.* ** *p* < 0.01 highlight significant growth differences by independent sample *t*-test.

To investigate the possibility that OMSV-Ch could be introduced into the virus-cured strain, the horizontal transmission potential of OMSV was trialed in co-culture by donor (OMSV-Ch-infected) and recipient (OMSV-Ch-cured) cultures, which were grown together to facilitate potential transfer of OMSV-Ch (Figure 6a). RT-PCR detection was conducted for OMSV-Ch and the result showed that recipient hyphae were positive for OMSV-Ch (Figure 6b). We also observed that the mycelia of the newly infected OMSV-Ch strain grew slower than the OMSV-Ch-cured strain at 5 days of incubation and this was significant according to *t*-test analysis (Figure 6c,d).

## 4. Discussion

According to the 10th ICTV report, fungal viruses with linear (+)ssRNA genomes are classified into seven families: *Betaflexiviridae*, *Gammaflexiviridae*, *Barnaviridae*, *Narnaviridae*, *Virgaviridae*, *Benyviridae*, and *Tymoviridae* (http://ictv.global/report/, accessed on 1 July 2021). The *Tymoviridae* family is currently comprised of three plant virus genera with a total of 40 species (*Tymovirus* 28, *Marafivirus* 11, and *Maculavirus* 1) and two unclassified species. In addition, several previously reported viruses may be members of the *Tymoviridae* family but have not yet been approved as such [30,31]. OMSV was first identified infecting oyster mushrooms in Korea. It has a single-stranded RNA genome and is most closely related to tymoviruses [26]. In this study, we identified the nearly complete nucleotide sequence of OMSV isolated from cultivated oyster mushrooms in China, thusly named OMSV-Ch. This strain shared the highest identity (74.9%) with OMSV from Korea. Based on phylogenetic analysis, OMSV is proposed to be a member of the *Tymoviridae* family. According to sequence comparisons, the two OMSV strains have greater divergence in the putative RP (84.1% identity) than in the conserved CP (94.1% identity). Phylogenetic analysis showed that the OMSV-Kr and OMSV-Ch were most closely related to *Tymoviridae* based on RP. However, these two strains formed a clade distinct from tymoviruses, marafiviruses, and maculaviruses based on the RP or CP, suggesting that the OMSV may belong to a new yet-to-be-established genus of the *Tymoviridae*.

As previously reported, *Tymoviridae* family members have a characteristic high cytidine content and the genome of OMSV-Ch also has a high cytosine proportion [30,31,32,33,34]. Nevertheless, the genomic size and structure of OMSV were different from the currently included three genera of the *Tymoviridae*. The OMSV genome size (5.7–5.8 kb) is smaller than those of tymoviruses (6.0–6.7 kb), marafiviruses (6.3–6.5 kb), or maculaviruses (7.5 kb). The OMSV genome does not contain a conserved “tymobox” (GAGUCUGAAUUGCUUC) or “marafibox” [CA(G/A)GGUGAAUUGCUUC] domains, which function as subgenomic RNA promoters in the genera *Tymovirus* and *Marafivirus*, respectively [32,35,36]. The fundamental biological difference is that OMSV is a mycovirus that infects mushrooms, while the members in the current three approved genera in the family *Tymoviridae* are all plant viruses [26,32,33]. There is evolutionary evidence that partitiviruses have been transmitted between plants and fungi [37,38]. It is currently not clear whether the OMSV could also infect plant hosts and this requires further study. Therefore, based on our results we propose that the OMSV belongs to a new genus and is tentatively named *Omsvirus* in the *Tymoviridae* family. Other biological properties of OMSV such as replication, movement, transmission, and the function of ORFs are unknown. Further work is needed to determine whether OMSV should be classified as a new member of the family *Tymoviridae* or of another family.

In order to investigate the effects of the mycovirus infection on its fungal host, virus-cured strains are commonly obtained using various methods, such as cycloheximide treatment, ribavirin treatment, hyphal tip isolation, protoplast regeneration, single-spore hybridization, heat treatment, or frozen and lyophilized treatments [39,40,41,42,43,44]. Previously, the dsRNA PoV-AS2792 was cured of the mycelia by the mycelial fragmentation combined with a single-colony isolation method [23]. In the OMIV and OMSV co-infected *P. ostreatus*, the two viruses were successfully eliminated by a successive transfer of mycelia to cAMP-rifamycin-containing plates [41]. A protoplast regeneration process was also used for the elimination of the POSV and other mycoviruses, which increased the yield by 18% in *P. ostreatus* TD300 [11]. In this study, we eliminated the OMSV-Ch from *P. ostreatus* via the cutting of hyphal tips combined with the high-temperature treatment method. The OMSV-Ch-cured and OMSV-Ch-infected isogenic strains with identical genetic backgrounds were used to investigate the effect of OMSV-Ch infection on *P. ostreatus*. Compared with the OMSV-Ch-cured strain, the OMSV-Ch-infected strain exhibited the following differences: (a) inhibited mycelial growth; (b) delayed fruiting body formation; (c) dysmorphic symptoms in the fruiting bodies; and (d) reduced yield of the fruiting bodies. The morphological and physiological changes in *P. ostreatus* caused by OMSV-Ch were similar to a dsRNA mycovirus (PoV-ASI2792) [23]. As the subcultures obtained after treatments with antibiotics might induce mutations in the genome, we back-introduced the OMSV-Ch into the virus-cured subculture via co-culture. The newly infected OMSV-Ch strain showed a lower growth rate compared with the isogenic OMSV-Ch-cured strain. Further studies are needed in order to explore the possible effects of OMSV-Ch on the morphology and yield of mushrooms by using these verified virus-cured and newly infected isogenic strains. Transcriptome analysis can be further used to identify the genes, processes, and pathways involved in OMSV infection, which will help us in further understanding the mechanisms of OMSV pathogenicity and contribute to the prevention and control of mycoviral diseases in the future.

## 5. Conclusions

In the current study, we present the molecular evidence that virus isolates from the *P. ostreatus* 8129 strain in China represent a new strain of OMSV, named OMSV-Ch. Sequence alignments between OMSV-Ch and OMSV-Kr showed an overall identity of 74.9 % at the nucleotide level. At the amino acid level, the virus exhibited 94.1 and 84.1% identities for the CP and RdRp, respectively. Phylogenetic analysis based on the RP showed that the OMSV-Ch clustered with OMSV-Kr, and the two strains formed a clade distinct from other tymoviruses, marafiviruses, and maculaviruses. These results suggest that the OMSV may belong to a new, yet-to-be-established genus of the *Tymoviridae*. Using single hyphal tip cultures combined with high-temperature treatment, we obtained the OMSV-Ch-cured *P. ostreatus* strain. Preliminary studies indicated that OMSV-Ch infection can significantly inhibit mycelial growth, cause malformation symptoms, and reduce the yield of fruiting bodies of the edible mushroom *P. ostreatus.* OMSV-Ch is transmitted horizontally to the OMSV-Ch-cured strain via hyphal contact. The newly infected OMSV-Ch mycelium exhibited slower growth than that of the OMSV-Ch-cured strain. This lays the groundwork for further study on the mechanisms of OMSV pathogenicity in the edible mushroom *P. ostreatus.*

## Figures and Tables

**Table 1:** Effects of the OMSV infection on the *P. ostreatus* measured in the cultivation test.

Strain	Period from Inoculation to Harvest (Day)	1st Flush Yield (g/bag)	2nd Flush Yield (g/bag)
OMSV-Ch-infected	35.36 ± 2.54	171.72 ± 11.45	121.10 ± 13.25
OMSV-Ch-cured	29.00 ± 1.67 ***	216.48 ± 14.92 ***	172.96 ± 9.18 ***

Data are the means ± standard deviations by independent sample *t*-test. *** *p* < 0.001; significant differences between OMSV-Ch-infected and OMSV-Ch-cured samples are marked with a star.

## Data Availability

The data presented in this study are available within the article.

## Supplementary Materials

The following are available online at https://www.mdpi.com/article/10.3390/biology11040574/s1, Figure S1: Microscopic observation of the mycelium diameters of the OMSV-Ch-infected and OMSV-Ch-cured *P. ostreatus* strains. Statistical analysis was performed using Student’s *t* test, Table S1: Sequences of primers used in this study.
