# Supplementary material for: Preliminary Studies on the Effects of Oyster Mushroom Spherical Virus China Strain on the Mycelial Growth and Fruiting Body Yield of the Edible Mushroom Pleurotus ostreatus
Source: Biology (Basel). 2022 Apr 10;11(4):574. doi: 10.3390/biology11040574 (PMC9029326; doi:10.3390/biology11040574)
Supplement: Supplementary file 1 [file biology-11-00574-s001.zip › biology-1614097-supplementary.pdf]

Table S1. Sequences of primers used in this study.

Primer	Sequence (5′-3′)	Note
OMSV-CPF	ATGTCTACTCCTACCCCCCAGGA	RT-PCR detection for OMSV [14]
OMSV-CPR	TCATATGACGCCGTATCCAAGTCC	
OMSV-4959F	TACCCCCCCAGGATCTCAAGCTTCT	RT-PCR detection for OMSV-Ch (Newly design)
OMSV-5605R	TGAAAGCGCGTCCATCAGAACCATTC	
OMSV-5F	CGTTGCTCACTGTGTCCCACTGCTCCCGGC	RT-PCR amplification for OMSV-Ch (Newly design)
OMSV-2888R	AGACCGCCGCAACTGGAAAA	
OMSV-2821F	GTCACGGCGCAGAACGCACG	RT-PCR amplification for OMSV-Ch (Newly design)
OMSV-3R	ATGTAAACCATATACCAAAC	
OMIV-1F	AACATTGTTGATCACGCTCT	RT-PCR detection for OMIV (Newly design)
OMIV-1R	GGCTTCAGAATAAAGATTGT	
PV-RDRPF	CCNNTNCAYTTTRYNGA	RT-PCR detection for PoV-ASI2792 [23]
PV-RDRPR	SWRTCNARNSWYTGNGT	
SN-F	ATAGAATTCAAAAATACTTGTCATCCC	RT-PCR detection for PoV-SN [24]
SN-R	ATAGAATTCCTAAACAAATAGACGTTG	
POSV-1F	AAACTCCTTGGTGCCCTCAA	RT-PCR detection for POSV (Newly design)
POSV-1R	ATTGTGAAAAAAGACTTGTA	
PoV1-F	ATCAAATCTTCCCTCTCCGA	RT-PCR detection for PoV1 (Newly design)
PoV1-R	CGGTAGGTATTGCGGATATT	
actin-F	AGTCGGTGCCTTGGTTAT	RT-qPCR detection for actin gene [28]
actin-R	ATACCGACCATCACACCT	

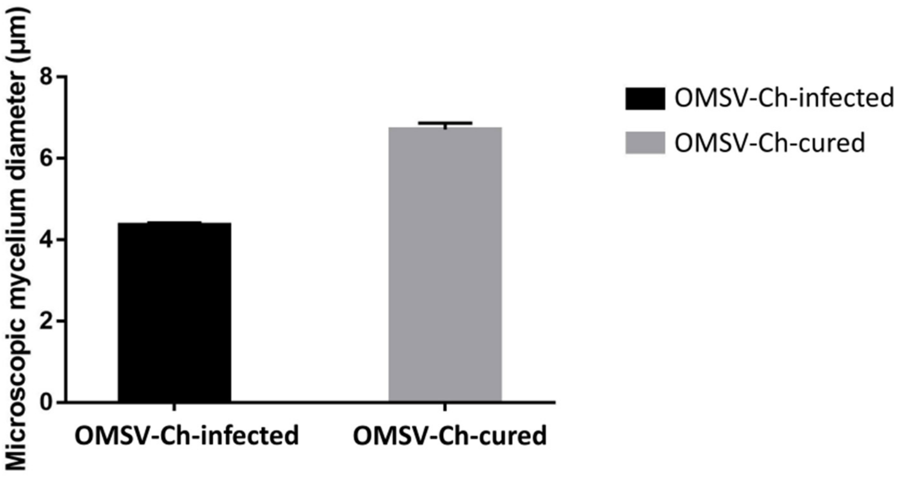

Figure S1. Microscopic observation of the mycelium diameters the OMSV-Ch-infected and OMSV-Ch-cured *P. ostreatus* strains. Statistical analysis was performed using Student's *t* test.
